# Supplementary material for: Progressive coevolution of the yeast centromere and kinetochore
Source: Nature. 2025 Nov 26;651(8107):1012–9. doi: 10.1038/s41586-025-09779-1 (PMC12925627; doi:10.1038/s41586-025-09779-1)
Supplement: Supplementary file 1 — Reporting Summary [file 41586_2025_9779_MOESM1_ESM.pdf]

nature portfolio

Corresponding author(s): Jana Helsen
Gavin Sherlock
Gautam Dey

Last updated by author(s): Oct 9, 2025

Reporting Summary

Nature Portfolio wishes to improve the reproducibility of the work that we publish. This form provides structure for consistency and transparency in reporting. For further information on Nature Portfolio policies, see our Editorial Policies and the Editorial Policy Checklist.

Statistics

For all statistical analyses, confirm that the following items are present in the figure legend, table legend, main text, or Methods section.

n/a	Confirmed	
☐	☒	The exact sample size (*n*) for each experimental group/condition, given as a discrete number and unit of measurement
☐	☒	A statement on whether measurements were taken from distinct samples or whether the same sample was measured repeatedly
☐	☒	The statistical test(s) used AND whether they are one- or two-sided<br>*Only common tests should be described solely by name; describe more complex techniques in the Methods section.*
☒	☐	A description of all covariates tested
☒	☐	A description of any assumptions or corrections, such as tests of normality and adjustment for multiple comparisons
☐	☒	A full description of the statistical parameters including central tendency (e.g. means) or other basic estimates (e.g. regression coefficient) AND variation (e.g. standard deviation) or associated estimates of uncertainty (e.g. confidence intervals)
☐	☒	For null hypothesis testing, the test statistic (e.g. *F*, *t*, *r*) with confidence intervals, effect sizes, degrees of freedom and *P* value noted<br>*Give P values as exact values whenever suitable.*
☒	☐	For Bayesian analysis, information on the choice of priors and Markov chain Monte Carlo settings
☒	☐	For hierarchical and complex designs, identification of the appropriate level for tests and full reporting of outcomes
☒	☐	Estimates of effect sizes (e.g. Cohen's *d*, Pearson's *r*), indicating how they were calculated

Our web collection on statistics for biologists contains articles on many of the points above.

Software and code

Policy information about availability of computer code

Data collection	ORFFinder Python (version 1.8), tblastn and pblast (BLAST suite version 2.13.0+, default parameters) were used to identify inner kinetochore proteins. PCAn and all other custom code related to this project can be found on GitHub: https://github.com/JHelsen/point-centromere-detection.
Data analysis	PCAn and all other custom code related to this project can be found on GitHub: https://github.com/JHelsen/point-centromere-detection. This includes code used for phylogenetic analyses and centromere transition simulations.<br>For PCAn, FIMO from the MEME suite v4.11.2, ORFFinder Python v1.8, and a local version of blastp v2.13.0+ were used.<br>For the protein evolutionary analyses, MAFFT v7.505, PAL2NAL v14, Gblocks v0.91b, aBSREL v2.5 and contrast-FEL v0.5 were used.<br>For Alphafold2 predictions and protein structure representations, Colabfold 1.5.5, AlphaFold2-multimer and UCSF ChimeraX v1.8 were used.

For manuscripts utilizing custom algorithms or software that are central to the research but not yet described in published literature, software must be made available to editors and reviewers. We strongly encourage code deposition in a community repository (e.g. GitHub). See the Nature Portfolio guidelines for submitting code & software for further information.

Data

Policy information about [availability of data](#)

All manuscripts must include a [data availability statement](#). This statement should provide the following information, where applicable:

- Accession codes, unique identifiers, or web links for publicly available datasets
- A description of any restrictions on data availability
- For clinical datasets or third party data, please ensure that the statement adheres to our [policy](#)

All data accompanying this manuscript can be found on FigShare: https://doi.org/10.6084/m9.figshare.c.7630151 (ref 72).
Genomes were downloaded from NCBI (https://www.ncbi.nlm.nih.gov/datasets/genome/), JGI (https://jgi.doe.gov), and from the supplemental data of four studies (References 48-51: doi: 10.1038/s41586-018-0030-5, doi: 10.1111/mec.13341, doi: 10.1016/j.gene.2024.148722, doi:10.1007/s00253-024-13267-3). Tables with all genome accession numbers (165 different species + 2737 S. cerevisiae isolates) can be found in the Supplementary Information and on FigShare: https://doi.org/10.6084/m9.figshare.c.7630151.
The TimeTree database (https://timetree.org) was used to retrieve the divergence time between S. cerevisiae and K. lactis.
The Yeast Gene Order Browser (http://ygob.ucd.ie) was used for synteny checks.
All materials are available upon request.

Research involving human participants, their data, or biological material

Policy information about studies with [human participants or human data](#). See also policy information about [sex, gender (identity/presentation), and sexual orientation](#) and [race, ethnicity and racism](#).

Reporting on sex and gender	NA
Reporting on race, ethnicity, or other socially relevant groupings	NA
Population characteristics	NA
Recruitment	NA
Ethics oversight	NA

Note that full information on the approval of the study protocol must also be provided in the manuscript.

Field-specific reporting

Please select the one below that is the best fit for your research. If you are not sure, read the appropriate sections before making your selection.

☒ Life sciences ☐ Behavioural & social sciences ☐ Ecological, evolutionary & environmental sciences

For a reference copy of the document with all sections, see [nature.com/documents/nr-reporting-summary-flat.pdf](#)

Life sciences study design

All studies must disclose on these points even when the disclosure is negative.

Sample size	No sample-size calculations were performed. Every Saccharomycetaceae species with available genome assembly and every available Saccharomyces cerevisiae genome assembly were included in the analysis.
Data exclusions	For the Saccharomyces cerevisiae intraspecific analyses, some strains were excluded from the main analysis: we removed duplicate strain backgrounds and limited our selection to 1,493 unique strains with phylogenetic information for the main figure. We did however annotate centromeres in the full set of strains (2,737) and the overall frequency of variant centromeres was very similar between the reduced and full dataset. No data was excluded from the plasmid loss experiments.
Replication	Plasmid loss assays were set up with at least 12 independent replicate populations. For each experiment, n is reported in the figure legend. The reproducibility of these experiments was very high.
Randomization	For population-level experiments, such as plasmid loss assays, randomization is not relevant as yeast cultures are uniform on a population level. All other analyses in this manuscript are computational and did not involve allocating samples into experimental groups.
Blinding	Most of the data were analyzed using semi-automated methods. During the experiments, blinding was not possible as each experiment was performed by an individual investigator who was aware of the experimental groups and treatments.

Reporting for specific materials, systems and methods

We require information from authors about some types of materials, experimental systems and methods used in many studies. Here, indicate whether each material, system or method listed is relevant to your study. If you are not sure if a list item applies to your research, read the appropriate section before selecting a response.

Materials & experimental systems

n/a	Involved in the study
☒	☐ Antibodies
☒	☐ Eukaryotic cell lines
☒	☐ Palaeontology and archaeology
☒	☐ Animals and other organisms
☒	☐ Clinical data
☒	☐ Dual use research of concern
☒	☐ Plants

Methods

n/a	Involved in the study
☒	☐ ChIP-seq
☒	☐ Flow cytometry
☒	☐ MRI-based neuroimaging

Plants

Seed stocks	NA
Novel plant genotypes	NA
Authentication	NA
